# Effects of cooking conditions on the physicochemical and sensory characteristics of dry- and wet-aged beef

**DOI:** 10.5713/ab.20.0852

**Published:** 2021-01-20

**Authors:** Dongheon Lee, Hyun Jung Lee, Ji Won Yoon, Minkyung Ryu, Cheorun Jo

**Affiliations:** 1 Department of Agricultural Biotechnology, Center for Food and Bioconvergence, and Research Institute of Agriculture and Life Science, Seoul National University, Seoul 08826, Korea; 2 Institute of Green Bio Science and Technology, Seoul National University, Pyeongchang 25354, Korea

**Keywords:** Beef, Cooking Method, Cooking Temperature, Dry Aging, Flavor, Wet Aging

## Abstract

**Objective:**

This study aimed to elucidate the effects of cooking conditions on the physicochemical and sensory characteristics of dry- and wet-aged beef strip loins.

**Methods:**

Dry- and wet-aged beef aged for 28 days were cooked using different cooking methods (grilling or oven roasting)×cooking temperatures (150°C or 230°C), and their pH, 2-thiobarbituric acid reactive substances (TBARS), volatile compounds, and color were measured.

**Results:**

Cooking conditions did not affect pH; however, grilling resulted in lower TBARS but higher cooking doneness at the dry-aged beef surface compared to oven roasting (p< 0.05). In descriptive sensory analysis, the roasted flavor of dry-aged beef was significantly stronger when grill-cooked compared to oven roasting. Dry-aged beef grill-cooked at 150°C presented a higher intensity of cheesy flavor, and that grilled at 230°C showed a greater intensity of roasted flavor compared to wet-aged beef at the same condition, respectively.

**Conclusion:**

Grilling may be effective for enhancing the unique flavor in dry-aged beef.

## INTRODUCTION

Dry aging is a method that exposes meat to controlled temperature, humidity, and air flow in the absence of packaging, in contrast to wet aging, which stores the meat vacuum-packaged [1]. In recent days, the consumer demand for dry-aged beef has been increasing, mainly due to its unique flavor, which is characterized as intensely roasted, beefy, and nutty [2]. It has been reported that dry-aged beef has higher quantities of flavor precursors, such as free amino acids and nucleotide-related compounds [3,4], and aromatic volatile compounds, such as aldehydes, compared to wet-aged beef [5].

On the other hand, meat flavor can be developed through the cooking process [6], and cooked meat sensory characteristics can vary depending on the cooking method (e.g., grilling, roasting, boiling, etc.) and conditions, including heating temperature and rate [7,8]. The influence of cooking methods on meat flavor has been observed in several studies to be mainly due to differences in the type of heat transfer (categorized as conduction, convection, and radiation) and the efficiency of heat treatment on meat [9–11]. Cooking can also be classified by cooking temperatures: low-temperature cooking below 100°C, high-temperature cooking above 100°C, and very high temperature cooking above 200°C [7]. Cooking at high temperatures increases the heating rate and the degree of Maillard reaction, which enhances the roasted aroma of meat. On the other hand, extensive cooking can cause high oxidative reactions and the generation of undesirable polycyclic aromatic hydrocarbons [8,12]. However, little is known about the effect of cooking method and temperature on dry-aged beef. King et al [13] applied oven roasting and microwave cooking to dry-aged beef and observed higher hydrocarbons and lower terpenoids in oven-roasted beef than in microwave-cooked beef. Nonetheless, as the authors stated, the lack of sensory evaluation restricts the estimation of the effect of cooking method on dry-aged beef. For this reason, the effective cooking method and temperature for dry-aged beef remain unclear.

Among the cooking methods for steaks in households and restaurants, grilling at temperatures above 200°C and oven roasting in the range of 150°C to 250°C are widely used conduction and convection cooking methods, respectively [8,12,14]. Therefore, the objective of this study was to investigate the effects of different cooking methods (grilling and oven roasting) and temperatures (150°C and 230°C) on the physicochemical and sensory characteristics of dry-beef.

## MATERIALS AND METHODS

### Sample preparation

#### Raw material and aging process

Beef strip loins (longissimus lumborum) from Holstein steers (21 months old, quality grade 3) were obtained at 48 h post-mortem. The visual fat and connective tissues were removed from the surface of beef strip loins, and each muscle was cut into an average weight of 500 g. Then, beef samples were randomly divided into two groups. One group was placed in a dry aging chamber (4°C, 75% relative humidity, and 2.5 m/s air flow velocity) and dry-aged for 28 days. The other group was vacuum packaged (HFV-600L, Hankook Fujee Machinery Co., Ltd., Hwaseong, Korea) in low-density polyethylene/nylon bags (oxygen permeability of 2 mL/m^2^/d at 0°C, 0.09 mm thickness; Sunkyung Co., Ltd., Seoul, Korea) for wet aging at 4°C, with the same aging duration. After the aging process, the dark and thickened crust of the dry-aged beef was trimmed off. Both dry- and wet-aged beef were stored in a vacuum packaged bag and frozen to −70°C for further analyses.

#### Cooking process

The beef strip loins were thawed at 4°C for 18 h and sliced into 3.5 cm thick samples (average weight of 100 g). Next, four different cooking conditions (cooking method × cooking temperature) were applied to the dry- (n = 3 for each cooking treatment) and wet-aged beef steaks (n = 3 for each cooking treatment): grilling at 150°C or 230°C, and oven roasting at 150°C or 230°C. Each cooking condition was replicated three times. During cooking, the surface temperature of the electric grill (EG-GW1700, Kitchenart, Incheon, Korea) was measured using an infrared thermometer (ST-101, Sincon, Bucheon, Korea), and the internal temperature of the sample was monitored using a digital thermometer (TM-747DU, Tenmars Electronics Co., Ltd., Taipei, Taiwan). Steaks cooked by grilling were turned every two min, and those cooked in an electric oven (MA324DBN, LG Electronics, Seoul, Korea) were turned at 40°C internal temperature. All cooking processes continued until the core temperature of the steak reached 72°C. In every cooking condition, dry-aged beef was compared with wet-aged beef, to which the same cooking treatment was applied, to determine whether the changes in meat sensory and physicochemical properties derived from the aging methods or cooking conditions.

### pH

One gram of meat sample with 9 mL of distilled water was homogenized at 9,600 rpm for 30 s using a homogenizer (T25 basic, IKA Works, Staufen, Germany). Then, the homogenates were centrifuged at 2,265×g for 10 min (Continent 512R, Hanil Co. Ltd., Daejeon, Korea), followed by filtration with filter paper (No. 4, Whatman International Ltd., Kent, UK). The pH values of dry- and wet-aged beef before and after cooking were measured using a pH meter (Seven2Go, Mettler-Toledo International Inc., Schwerzenbach, Switzerland). The pH meter was pre-calibrated with pH 4.01, pH 7.00, and pH 9.21 standardized buffer solutions at room temperature.

### Lipid oxidation

Lipid oxidation was determined by measuring 2-thiobarbituric acid reactive substance (TBARS) values. Each sample (5 g) was homogenized at 9,600 rpm for 30 s using a homogenizer (T25 basic, IKA works, Germany), with the addition of 15 mL of distilled water and 50 μL of 7.2% butylated hydroxy toluene solution. After centrifugation at 2,265×g for 15 min (Continent 512R, Hanil Co. Ltd., Korea), the supernatants were filtered using filter paper (No. 4, Whatman International Ltd., UK). Then, 2 mL of the filtrates was transferred to a test tube and mixed with 4 mL of 20 mM 2-thiobarbituric acid in 15% trichloroacetic acid. The mixture was heated in a water bath at 90°C for 30 min, cooled, and centrifuged at 2,265×g for 15 min (Continent 512R, Hanil Co. Ltd., Korea). The supernatant absorbances were measured at 532 nm using a spectrophotometer (X-ma 3100, Human Co. Ltd., Seoul, Korea). TBARS values were expressed as mg malondialdehyde per kg of meat sample.

### Volatile compound analysis

The analysis of volatile compounds in cooked beef was performed by solid-phase microextraction and gas chromatography-mass spectrometry (SPME-GC-MS). Five grams of cooked meat samples were placed into a 20-mL headspace vial and sealed with a PTFE-faced silicone septum. The samples were incubated at 40°C for 5 min, and then, a 65 μm thick polydimethylsiloxane/divinylbenzene fiber (Supelco Inc., Bellefonte, PA, USA) was exposed to the headspace of the vial for 60 min. The volatile compounds were desorbed in the injection port of the GC (Trace 1310, Thermo Fisher Scientific, Waltham, MA, USA) at 270°C in splitless mode. Helium was used as the carrier gas at a flow rate of 2 mL/min, and volatile compounds were separated using a fused silica capillary column (DB-Wax, 60 m×0.25 mm i.d., and 0.50 μm film thickness; Agilent Technologies Inc., Santa Clara, CA, USA). The GC oven was programmed as follows: initial temperature of 40°C, subsequently increased to 180°C at a rate of 5°C/min, then increased to 200°C at a rate of 2°C/min and held for 5 min, and then increased to a final temperature of 240°C at a speed of 10°C/min and held for 10 min. The column was directly coupled to a triple quadrupole mass spectrometer (TSQ 8000, Thermo Fisher Scientific, USA) operating in the electron ionization mode at 70 eV and 250°C. Mass spectra were obtained with a scan ranging from 35 to 550 m/z at intervals of 0.2 s. The identification of volatile compounds was performed by comparing their mass spectra with those of the National Institute of Standards and Technology (NIST) mass spectral library.

### Meat color

The cooked meat was cut horizontally to measure its surface and internal color. Meat color was measured using a colorimeter (CM-5, Konica Minolta Censing Inc., Osaka, Japan), which was calibrated using a standard plate before measurements. The CIE L*- (lightness), a*- (redness), and b*- (yellowness) values were determined in the condition of illuminant D65 and 10° standard observer with a 30 mm aperture size plate. A reflectance ratio of 630:580 nm was calculated to estimate the degree of doneness after different cooking treatments [15].

### Descriptive sensory analysis

The design of the descriptive sensory analysis for dry- and wet-aged beef was reviewed and approved by the Institutional Review Board (IRB) of Seoul National University (SNU) (IRB No. 1810/003-001). Immediately after cooking, the samples were cut to 1 cm in thickness, wrapped in aluminum foil and plastic wrap to preserve the aroma and prevent moisture evaporation, and kept in a drying oven (BF-80N, BioFree, Seoul, Korea) at 60°C. The holding time of the cooked samples in the drying oven was less than 20 min. Ten trained panelists (6 males and 4 females aged 26 to 33 years) were recruited from SNU researcher and faculty populations, and the panelists participated in the descriptive sensory analysis. Before the analysis, panelists were trained over several sessions for the descriptive sensory analysis of dry- and wet-aged beef and practiced rating the score of each sensory attribute. All training sessions and descriptive sensory analyses were conducted at SNU. The sensory properties were evaluated using a nine-point hedonic scale, in which the flavor scores ranged from one to nine (extremely weak to extremely strong), the score of surface color ranged from one to nine (extremely bright to extremely dark), the internal color score ranged from one to nine (extremely white to extremely red), the tenderness score ranged from one to nine (extremely tender to extremely tough) after 15 bites, and the score of juiciness ranged from one to nine (extremely dry to extremely juicy) after 15 bites. Drinking water was provided to the panelists to cleanse their palates between sample evaluations.

### Statistical analysis

All experiments were conducted in triplicate, except the descriptive sensory analysis, where a randomized block design was applied using the trial and panelist as the block (n = 10 per trial, 20 per 2 trials). Statistical analysis was performed using the general linear model (SAS 9.4, SAS Institute Inc., Cary, NC, USA), which included the aging method, cooking condition, and their interactions as fixed effects and carcass and carcass side as random effects. For the evaluation of descriptive sensory analysis data, the trial and panelist were also included as random factors. The results were reported as mean values with standard error of the mean, and significant differences among the mean values were determined by the Tukey’s multiple comparison test at a significance level of 0.05. In order to identify the difference in the composition of volatile compounds between treatments and classify them, principal component analysis (PCA), partial least squares-discriminant analysis (PLS-DA), and variable importance in projection (VIP) scores for the PLS-DA model were performed with the contents of volatile compounds using MetaboAnalyst 4.0 (www.metaboanalyst.ca) according to Kim et al [1], and the samples were log-transformed and auto-scaled before conducting multivariate analyses. Pearson correlation coefficients and linear mixed model between sensory properties and overall acceptability of dry- and wet-aged beef strip loins were analyzed using SAS 9.4 (SAS Institute Inc., USA). In the mixed model, random terms included the trial and panelist. The model is as follows:

$$\begin{array}{l} \text{Overall acceptability}\\ =\text{surface color}+\text{internal color}+\text{roasted flavor}\\ +\text{dry-aged flavor}+\text{cheesy flavor}+\text{fatty flavor}\\ +\text{savory flavor}+\text{tenderness}+\text{juiciness}+\text{trial}+\text{panelist.} \end{array}$$

## RESULTS

### pH

The pH values of dry- and wet-aged beef differed before cooking (5.57 and 5.27, respectively; p<0.05). Similarly, it was reported that dry-aged beef showed significantly higher pH compared to wet-aged beef after 21 or 40 days of aging [3,16]. The pH difference between dry- and wet-aged beef could be due to their different microbiological compositions [2]. Various environmental factors such as temperature, relative humidity, air flow velocity, and the presence of oxygen affect the growth of microorganisms [4]. Higher degrees of total aerobic bacteria and mold and yeast counts were found in dry-aged beef, while lactic acid bacteria was more dominant in wet-aged beef after 14 and 21 days of aging [17]. Notably, the proteolytic and lipolytic effect of mold and yeast on dry-aged beef was suggested in previous studies [4,18]. As a result, the formation of ammonia, amines, and basic amino acids by proteolysis might lead to the increase of the pH of dry-aged beef. On the other hand, the accumulation of lactic acid by the increase of lactic acid bacteria in wet-aged beef could decrease its pH [3]. Dry-aged beef also had a higher pH than wet-aged beef after cooking (p<0.05; Table 1). According to Kerth and Miller [6], an increase in pH can lead to an increase in the water holding capacity, which affects heat transfer efficiency and ultimately results in the flavor of meat. They stated that the increase in beef surface temperature with low water holding capacity during cooking is disturbed by the evaporation of free water on the surface, which leads to the formation of fewer Maillard reaction products that are associated with meaty and roasted aroma. Furthermore, it was reported that the formation of specific Maillard reaction products such as pyrazines was favored as the pH of meat increased between 4.5 and 6.5 [19]. Madruga and Mottram [20] measured volatile compounds in cooked meat at pH values ranging from 4.0 to 5.6, and observed a decrease in 2-methyl-3-furanyl group compounds and sulfur compounds and the increase of pyrazines as the pH of the meat increased. In the present study, we also detected a significant increase in pyrazine compounds in dry-aged beef compared to wet-aged beef and discussed the results below. However, the effects of cooking method and cooking temperature on pH were not observed in this study.

### Lipid oxidation

During the cooking process, lipid oxidation has a huge role in the generation of desirable and characteristic flavor compounds in meat, although excessive lipid oxidation leads to the deterioration of meat quality, such as undesirable off-flavor and texture changes [10,21].

The TBARS value of dry-aged beef was significantly lower than that of wet-aged beef, regardless of cooking conditions (Table 2). This finding was inconsistent with the results from the study conducted by Ribeiro et al [22], who reported that the TBARS value of beef loin was significantly higher after dry aging for 42 days compared to that after wet aging for the same duration. The increase of lipid oxidation in dry-aged beef may be related with air exposure during aging process, while lipid oxidation is prohibited in vacuum-packed wet-aged beef [22]. In the present study, the lower TBARS value of cooked dry-aged beef might be related to an increase in antioxidant compounds in dry-aged meat, as reported in previous studies [1,18,23]. Kim et al [1] found that after 28 days of aging, the concentrations of anserine, carnosine, and aromatic amino acids, compounds with strong antioxidant activities, were significantly higher in dry-aged beef than in wet-aged beef. Lee et al [4] also reported higher amounts of amino acids, including phenylalanine, tryptophan, and tyrosine, in dry-aged beef compared to wet-aged beef, which could result from concentration effects due to moisture evaporation and microbial proteolysis during the dry aging process. Moreover, Park et al [23] observed that in dry-aged beef patties made with 5% crust, the surface of dry-aged beef, which is usually trimmed off, showed lower TBARS values compared to those made without crust, and suggested potential antioxidant activity in the crust. The antioxidant activities of dry- and wet-aged beef and crust from dry-aged beef were compared by Choe et al [18] through radical scavenging activities, ferric ion reducing capacity, and metal chelating activity tests. In this study, the investigators found that dry-aged beef possessed higher antioxidant activity than wet-aged beef, and crust showed the highest antioxidant activity. This antioxidant activity in dry-aged beef, especially in the crust, might be attributed to the increase of small peptides (<3 kDa) through the action of microbial enzymes in the crust [18].

Meanwhile, the TBARS value of oven-roasted beef strip loin was significantly higher than that of grilled beef at both cooking temperatures (p<0.05). Moreover, steaks cooked at lower temperatures showed higher TBARS values than those cooked at higher temperatures, even though there was no significant difference between grilled dry-aged beef cooked at different temperatures. It was reported that the TBARS value could be affected by the cooking temperature and cooking time [9,21]. Broncano et al [21] found that the TBARS value of Iberian pork roasted at 150°C for 20 min was significantly higher than that of pork grilled at 190°C for 4 min. Domínguez et al [9] also observed that roasting of foal meat at 200°C for 12 min produced more oxidation compared to grilling at 130°C to 150°C for 5 min on each surface. In this study, we observed that grilling required less cooking time compared to oven roasting (13 min 14 s and 21 min 7 s, respectively; p<0.05). The correlation between the TBARS value and cooking time was found to be significant (r^2^ = 0.64; p< 0.01).

Accordingly, the lower TBARS values in dry-aged beef compared to those in wet-aged beef in the present study might result from the increase of bioactive peptides and antioxidants by the action of mold and yeast as described above. Furthermore, prolonged cooking process would accelerate the lipid oxidation, which might negatively affect the sensory quality of beef.

### Volatile compound analysis

**Figure 1:**
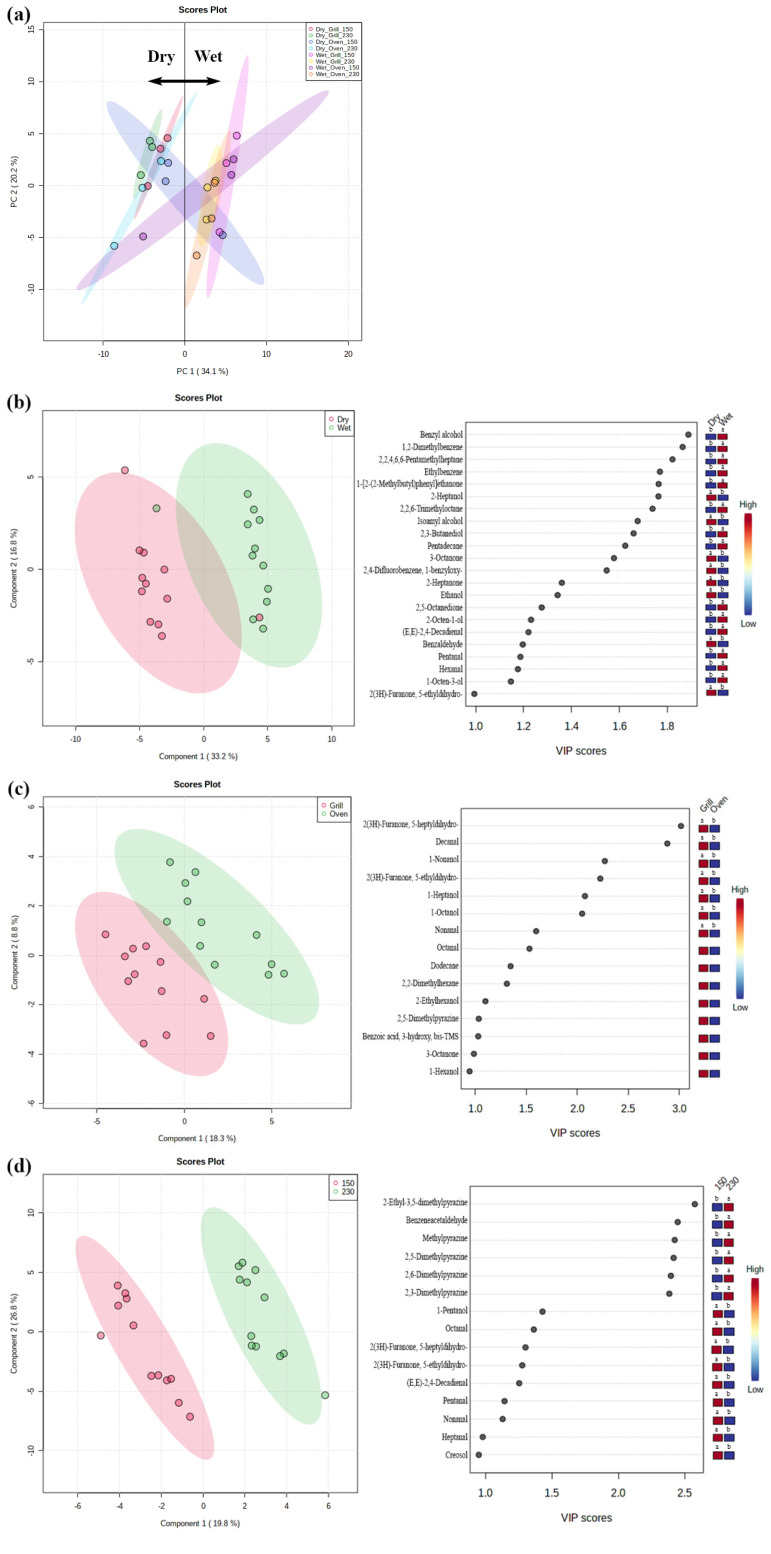
Principal component analysis (PCA) of dry- and wet-aged beef strip loins cooked at different cooking conditions (a) and partial least squares-discriminant analysis (PLS-DA) and its variable importance in projection (VIP) scores from dry- and wet-aged beef (b), grilled and oven-roasted beef (c), and beef cooked at 150°C and 230°C (d). The colored box next to the PLS-DA VIP scores represents the relative concentration of each volatile compound (red, high; blue, low). Dry, dry-aged beef; Wet, wet-aged beef; Grill, grill-cooked; Oven, oven-cooked; 150, cooked at 150°C; 230, cooked at 230°C. Different letters within the same row indicate significant differences (p<0.05). Principal component analysis (PCA) of dry- and wet-aged beef strip loins cooked at different cooking conditions (a) and partial least squares-discriminant analysis (PLS-DA) and its variable importance in projection (VIP) scores from dry- and wet-aged beef (b), grilled and oven-roasted beef (c), and beef cooked at 150°C and 230°C (d). The colored box next to the PLS-DA VIP scores represents the relative concentration of each volatile compound (red, high; blue, low). Dry, dry-aged beef; Wet, wet-aged beef; Grill, grill-cooked; Oven, oven-cooked; 150, cooked at 150°C; 230, cooked at 230°C. Different letters within the same row indicate significant differences (p<0.05).

A total of 60 volatile compounds, including 15 alcohols, 10 aldehydes, 15 aliphatic hydrocarbons, 12 aromatic hydrocarbons, 6 ketones, and 2 unclassified compounds were identified in the headspace of cooked dry- and wet-aged beef. The PCA showed that the volatile profiles of dry- and wet-aged beef differed, except for those of oven-roasted samples at 150°C (Figure 1a). Similarly, the PLS-DA plot distinguished dry- and wet-aged beef, and we found that dry-aged beef had higher 2-heptanol, isoamyl alcohol, 3-octanone, 2-heptanone, and benzaldehyde concentrations, whereas wet-aged beef had more abundant benzyl alcohol, 1,2-dimethylbenzene, 2,2,6-trimethyloctane, 2,5-octanedione, and 2,3-butanediol species (Figure 1b). The aforementioned compounds were regarded as the most characteristic variables for the separation of the two groups, and the alcohol, aldehyde, aliphatic hydrocarbon, and ketone variables represent lipid oxidation-derived products [9]. In particular, aldehydes and ketones contribute highly to cooked meat flavor because they have low odor threshold values [5,10]. The difference in the concentration of lipid oxidation-derived products might result from the different susceptibilities of dry- and wet-aged beef to lipid oxidation (Table 2). Kim et al [3] reported differences in the composition of free fatty acids and free amino acids between dry- and wet-aged beef, and Lee et al [4] observed that dry aging for 28 days was more effective in increasing free amino acids and reducing sugars compared to wet aging for the same duration. It seems that different flavor precursors might influence the volatile formation of cooked dry- and wet-aged beef.

The effect of cooking method on the formation of volatile compounds was detected using the PLS-DA model (Figure 1c). Most volatile compounds with VIP scores higher than one were more abundant in grilled beef than in oven-roasted samples. This observation could be related to the surface temperature of the samples and the efficacy of heat transfer depending on the cooking method. Peñaranda et al [11] reported that the intensity of meat odor was higher in grill-cooked pork compared to oven-roasted pork, possibly because of the higher surface temperature. Silva et al [24] found significantly higher amounts of Maillard reaction products in grilled and fried jerky chicken than in oven-roasted and sous-vide cooked chicken, and stated that conduction cooking was more effective in heat transfer than convection cooking.

Finally, noticeable changes were found in five pyrazines (2-ethyl-3,5-dimethylpyrazine, 2,3-, 2,5-, and 2,6-dimethylpyrazine, and methylpyrazine) as cooking temperature increased (Figure 1d). This observation was in accordance with the study conducted by Wall et al [25], where the production of pyrazines in beef steak increased with increasing grill surface temperature from 177°C to 232°C. Pyrazines are mainly derived from the Maillard reaction, which requires high temperatures above 110°C in meat [26], and the formation of these compounds increases at elevated surface temperatures [24]. Yoo et al [27] reported that the searing of beef steaks at 250°C increased meaty and roasted aromas compared to oven-cooking 180°C, due to the increased occurrence of the Maillard reaction. Furthermore, 2,3- and 2,5-dimethylpyrazine and methylpyrazine were present in significantly higher quantities in dry-aged beef when cooked by grilling compared to wet-aged beef (data not shown). Ha et al [5] also observed higher abundances of pyrazine, methylpyrazine, 2,5-dimethylpyrazine, 2-ethylpyrazine, and 2,5-dimethyl-3-ethylpyrazine in dry-aged beef compared to wet-aged beef. Pyrazines have meaty, nutty, and roasted aroma flavors. The higher concentration of pyrazine compounds in dry-aged beef may contribute to the development of characteristic dry-aged flavor [10].

### Meat color

The color of cooked meat is attributed to the heat-induced denaturation of myoglobin, which results in a brown appearance [28]. Meat color can be influenced by various factors, such as pH, cooking conditions, the chemical state of myoglobin, and other variables [15]. In order to analyze the effect of cooking conditions on cooked meat color in depth, both the surface and internal meat colors were measured independently. As a result, the beef surface color was generally affected by cooking conditions rather than by the aging method (Table 3). Noticeably, we found that L*-, a*-, and b*- values were significantly lower at the surface of grill-cooked steaks at 230°C compared to other treatment combinations, regardless of the aging method. In both dry- and wet-aged beef, oven roasting led to a brighter surface color compared to grill cooking at the same cooking temperature (p<0.05). Moreover, the b*-value was significantly higher at the surface of oven-roasted beef compared to that of grilled beef when cooked at 230°C. In the case of cooking temperature, lower temperature cooking generally led to higher L*-, a*-, and b*-values of the beef surface compared to higher temperature cooking. Lower L*-values due to grilling or higher-temperature cooking might be related to moisture loss and surface drying due to a higher meat surface temperature [22]. The decrease in redness that was observed as cooking temperature increased from 150°C to 230°C indicates that myoglobin denaturation occurred to a higher degree at higher cooking temperatures [28]. Regarding yellowness, Mitacek et al [29] stated that the increase in metmyoglobin might be related to a decrease in yellowness. Consequently, grill-cooked dry- and wet-aged beef showed a higher degree of doneness than oven-cooked dry-aged beef at 230°C and oven-cooked wet-aged beef at 150°C, respectively.

On the other hand, all internal beef steak color parameters showed significant differences depending on the aging method. In general, L*-, a*-, and b*-values were higher and the degree of doneness was lower in wet-aged beef than in dry-aged beef. It has been reported that the surface of dry-aged beef had lower L*- and a*-values than that of wet-aged beef [16,22]; however, the internal color of cooked dry- and wet-aged beef has been rarely compared. The differences in beef color stability might be attributed to lipid oxidation, reducing ability, oxygen consumption rate, or the composition of three forms of myoglobin [22,28]. In the case of cooking conditions, oven roasting at 230°C showed significantly higher internal meat color L*- and b*-values compared to grilling at the same cooking temperature. While the internal color of grill-cooked dry-aged beef at 230°C showed overall low a*- and b*-values and a high degree of doneness, grilled dry-aged beef at 150°C had the highest a*- and b*-values and the lowest degree of doneness among the four cooking conditions. Yancey et al [14] observed the effect of cooking method on the internal cooked color of meat and reported that the conduction cooking method denatured myoglobin to a greater extent, resulting in a less red appearance compared to oven cooking. In accordance with the surface color results, the high abundance of pyrazine compounds in grilled dry-aged beef at 230°C could be explained by the high degree of doneness estimated by the surface and internal color measurements.

Meat color can provide information about the eating quality of meat to consumers [8]. For example, browned surface color can be utilized as an indicator of the Maillard reaction and caramelization, and the internal cooked color can indicate the doneness of meat [27,28]. The degree of doneness was further evaluated through a descriptive sensory analysis, as discussed below.

### Descriptive sensory analysis

Grill-cooked steak had a darker surface color compared to oven roasted steak, except for wet-aged beef cooked at 150°C (Table 4). However, there was no difference in internal redness between grilled and oven-roasted beef steaks cooked at the same temperature. Considering the results from the instrumental color measurements (Table 3) and descriptive sensory analysis, the aged beef color was more likely to be affected by cooking conditions than aging method, and grilling at higher temperatures was more desirable for the cooked meat color.

In the dry-aged beef, grill-cooked steaks showed no difference in color, flavor, tenderness, or juiciness scores (Table 4). Grilling had a significantly higher score for roasted flavor in dry- and wet-aged beef at both cooking temperatures compared to oven roasting. This result supports the results of the volatile compound analysis and meat color, where grilling was more effective for producing desirable flavor compounds and increasing cooking doneness (efficiency of heat transfer). In case of tenderness, no effect of cooking condition was found within dry-aged beef, while in wet-aged beef grilling at 150°C led to significantly lower shear force. Wall et al [25] observed no difference in the shear force of grill-cooked beef by surface temperature of grill (177°C, 205°C, and 232°C), whereas Yancey et al [14] found that conduction cooking resulted in a higher shear force than convection cooking. The juiciness score was higher in grill-cooked dry-aged beef at the cooking temperature of 230°C compared to oven-roasted dry-aged beef. In general, the juiciness is influenced by the amounts of moisture in meat after cooking [8,11]. In the result of present study, the surface of grilled dry-aged beef showed higher degree of doneness compared to that of oven-roasted one cooked at 230°C. During grilling or roasting, crust can be formed at the dried surface of meat [27] which might reduce the moisture permeability [30]. If higher heat transfer efficiency of grilling is considered than oven roasting in this study, crust of beef may potentially help increase the juiciness.

Based on the Pearson correlation analysis, the meat color, roasted and savory flavor, and juiciness were strongly related to the overall acceptability of both aged beef (Table 5). The relationships between internal meat color and roasted flavor with overall acceptability were also significant, respectively, when using linear mixed model. As the brown surface color indicates the degree of caramelization and the point of consumption [27,28], the positive correlation between the degree of doneness and overall acceptability is natural. The sensory scores of meat color and roasted flavor suggest that grilling instead of oven roasting would be effective for dry-aged beef (Table 4). Moreover, dry-aged beef that was grill-cooked at 150°C had significantly higher scores for surface color, dry-aged and cheesy flavor compared to wet-aged beef. We found that cheesy and savory flavors are positively correlated (r^2^ = 0.53; p<0.05). This indicates that the unique flavor of dry-aged beef could be perceived strongly even at lower cooking temperature and might be more attractive for consumers who prefer dry-aged and cheesy flavors than that of wet-aged beef after grilling. The flavor difference between dry- and wet-aged beef was not observed (except the dry-aged flavor) only when the dry-aged beef was grill-cooked at 150°C while the wet-aged beef was grilled at much higher cooking temperature (p>0.05). This range of cooking temperature for dry-aged beef (150°C) has benefits for reducing overcooking and increases of heterocyclic aromatic amines and polycyclic aromatic hydrocarbons compared to the higher cooking temperature for wet-aged beef (230°C) [12]. Meanwhile, an obvious contrast between the roasted flavor of dry- and wet-aged beef was observed when both samples were grill-cooked at 230°C. As discussed above, higher pyrazine compound concentrations in dry-aged beef compared to wet-aged beef could intensify the roasted flavor of dry-aged beef. Although no difference in cheesy flavor was found between them (p> 0.05), the characteristic flavor of dry-aged beef compared to wet-aged beef could be derived from the significantly higher intensities of roasted and dry-aged flavor. As roasted flavor was positively correlated with overall acceptability of beef (Table 5), grilling at higher cooking temperature could also be effective for the purpose of dry aging to develop the desirable beef flavor.

From the results, grilling of dry-aged beef at both lower temperature (150°C) and higher temperature (230°C) had their own advantages. The former led to higher intensities of surface color and cheesy flavor of dry-aged beef compared to those of wet-aged beef cooked at the same temperature. The latter presented a higher roasted flavor than wet-aged beef without any perceivable sensory defects. Consequently, grill cooking at both 150°C and 230°C might be promising cooking conditions for dry-aged beef to obtain the characteristic flavors and acceptance by a wide variety of consumers.

## CONCLUSION

In conclusion, the advantages of dry aging can be enhanced by grill cooking instead of oven roasting, as grilling improves desirable flavor and color. In addition, the grill-cooked dry-aged beef might be appealing to consumers due to its intense roasted flavor, compared to grill-cooked wet-aged beef at the same cooking condition, and it is greater when cooking temperature is higher. Within the treatments in this study, grill cooking of dry-aged beef at a higher temperature (230°C) would be recommended.

## Figures and Tables

**Table 1:** Effects of different cooking conditions on pH of dry- and wet-aged beef strip loins

Aging method	Grilled		Oven-roasted		SEM^1)^
	
	150°C	230°C	150°C	230°C	
Dry	5.83^x^	5.84^x^	5.82^x^	5.82^x^	0.011
Wet	5.42^y^	5.43^y^	5.45^y^	5.43^y^	0.012
SEM^2)^	0.008	0.010	0.016	0.008	-

SEM, standard error of the mean.

1) n = 12.

2) n = 6.

x,y Different letters within the same column indicate statistically significant differences (p<0.05).

**Table 2:** Effects of different cooking conditions on lipid oxidation (mg malondialdehyde/kg meat) of dry- and wet-aged beef strip loins

Aging method	Grilled		Oven-roasted		SEM^1)^
	
	150°C	230°C	150°C	230°C	
Dry	1.44^cy^	1.55^cy^	2.36^ay^	1.91^by^	0.076
Wet	2.98^cx^	2.43^dx^	4.59^ax^	4.06^bx^	0.149
SEM^2)^	0.157	0.137	0.075	0.083	-

SEM, standard error of the mean.

1) n = 12.

2) n = 6.

a,b,c,d Different letters within the same row indicate statistically significant differences (p<0.05).

x,y Different letters within the same column indicate statistically significant differences (p<0.05).

**Table 3:** Effects of different cooking conditions on color of dry- and wet-aged beef strip loins

Trait	Aging method	Grilled		Oven-roasted		SEM^1)^
	
		150°C	230°C	150°C	230°C	
		----------------------------------------------- Surface ------------------------------------------------------				
*L* ^*^	Dry	37.19^bx^	29.94^c^	39.97^ax^	38.42^by^	0.314
	Wet	34.03^c^^y^	30.63^d^	38.97^by^	42.02^ax^	0.447
	SEM^2)^	0.370	0.616	0.239	0.149	-
*a* ^*^	Dry	6.28^a^	5.18^b^	6.31^ay^	6.76^a^	0.157
	Wet	6.55^b^	6.03^b^	7.42^ax^	6.50^b^	0.170
	SEM^2)^	0.129	0.276	0.086	0.084	-
*b* ^*^	Dry	10.22^a^	3.93^by^	10.40^ax^	11.00^ay^	0.391
	Wet	8.69^b^	5.97^cx^	9.34^by^	12.64^ax^	0.165
	SEM^2)^	0.493	0.290	0.125	0.133	-
Degree of doneness^3)^	Dry	1.28^b^	1.27^b^	1.31^aby^	1.34^a^	0.011
	Wet	1.29^b^	1.29^b^	1.45^ax^	1.29^b^	0.011
	SEM^2)^	0.006	0.014	0.007	0.014	-
		---------------------------------------------------- Internal -----------------------------------------------------				
*L* ^*^	Dry	49.05^by^	51.64^a^	52.07^ay^	50.28^aby^	0.423
	Wet	51.94^c^^x^	52.88^b^	55.06^ax^	54.71^ax^	0.186
	SEM^2)^	0.150	0.425	0.207	0.425	
*a* ^*^	Dry	12.35^a^	6.06^by^	6.83^by^	6.97^by^	0.476
	Wet	13.39^a^	9.21^bx^	12.78^ax^	13.24^ax^	0.482
	SEM^2)^	0.653	0.553	0.320	0.290	-
*b* ^*^	Dry	16.16^ay^	13.28^c^^y^	12.81^d^^y^	14.00^by^	0.091
	Wet	17.09^ax^	15.91^bx^	17.38^ax^	17.60^ax^	0.191
	SEM^2)^	0.129	0.173	0.148	0.144	-
Degree of doneness	Dry	2.57^a^	1.43^by^	1.53^by^	1.50^by^	0.101
	Wet	2.74^a^	1.90^bx^	2.59^ax^	2.66^ax^	0.087
	SEM^2)^	0.141	0.096	0.051	0.062	-

SEM, standard error of the mean.

1) n = 12.

2) n = 6.

3) The ratio of reflectance of light at 630 nm and 580 nm, which indicates the degree of doneness [15].

a,b,c,d Different letters within the same row indicate statistically significant differences (p<0.05).

x,y Different letters within the same column indicate statistically significant differences (p<0.05).

**Table 4:** Descriptive sensory analysis on dry- and wet-aged beef strip loins cooked at different cooking conditions

Trait	Aging method	Grilled		Oven-roasted		SEM^1)^
	
		150°C	230°C	150°C	230°C	
		----------------------------------------------------- Color ---------------------------------------------------------				
Surface	Dry	6.85^a^^x^	7.25^a^	5.75^b^	5.20^b^	0.215
	Wet	5.75^b^^y^	6.85^a^	5.55^b^	5.40^b^	0.217
	SEM^^2)^^	0.286	0.192	0.213	0.151	
Internal	Dry	4.85^ab^	4.85^ab^	5.05^a^	4.30^b^	0.319
	Wet	5.30	4.90	4.40	4.65	0.407
	SEM^2)^	0.502	0.348	0.312	0.253	
		------------------------------------------------------- Flavor ----------------------------------------------------------				
Roasted	Dry	6.40^a^	7.20^a^^x^	5.25^b^	5.10^b^	0.281
	Wet	5.85^a^	6.10^a^^y^	4.75^b^	4.95^b^	0.294
	SEM^2)^	0.345	0.216	0.279	0.296	
Dry-aged	Dry	4.75	4.95	5.95	5.10	0.374
	Wet^3)^	-	-	-	-	-
Cheesy	Dry	5.00^x^	4.25	5.45^x^	4.15	0.384
	Wet	2.65^y^	3.65	2.85^y^	2.95	0.366
	SEM^2)^	0.321	0.417	0.321	0.427	
Fatty	Dry	4.20	4.40	4.70	3.95	0.392
	Wet	4.30	4.20	3.65	4.50	0.360
	SEM^2)^	0.371	0.363	0.404	0.366	
Savory	Dry	5.30	5.55	5.75	5.45	0.345
	Wet	5.60	5.45	4.50	4.65	0.402
	SEM^2)^	0.342	0.290	0.468	0.376	
		--------------------------------------------------------- Texture ---------------------------------------------------------				
Tenderness	Dry	5.55^x^	5.85	6.45^x^	5.80	0.326
	Wet	3.65^b^^y^	5.35^a^	5.15^a^^y^	4.85^a^	0.367
	SEM^2)^	0.294	0.317	0.349	0.417	
Juiciness	Dry	3.90^ab^^y^	4.35^a^	3.70^ab^	2.95^b^^y^	0.312
	Wet	5.30^x^	4.05	4.10	4.20^x^	0.403
	SEM^2)^	0.373	0.390	0.340	0.334	

The descriptive sensory analysis was performed twice, and the result was analyzed using a randomized block design with 10 panelists per trial.

SEM, standard error of the mean.

1) n = 8.

2) n = 4.

3) Not tested as dry-aged flavor cannot exist in wet-aged beef.

a,b Different letters within the same row indicate statistically significant differences (p<0.05).

x,y Different letters within the same column indicate statistically significant differences (p<0.05).

**Table 5:** Pearson correlation coefficients and regression coefficients by linear mixed model between sensory properties and overall acceptability of dry- and wet-aged beef strip loins

Coefficient	Surface color	Internal color	Roasted flavor	Dry-aged flavor	Cheesy flavor	Fatty flavor	Savory flavor	Tenderness	Juiciness
Correlation	0.55^*^	0.58^*^	0.60^*^	ns	ns	ns	0.61^*^	ns	0.54^*^
Regression	ns	0.17^*^	0.26^**^	ns	ns	ns	ns	ns	ns

* p<0.05;

** p<0.01.

ns, not significant (p>0.05).
